# Food Safety in the European Union: A Comparative Assessment Based on RASFF Notifications, Pesticide Residues, and Food Waste Indicators

**DOI:** 10.3390/foods14142501

**Published:** 2025-07-17

**Authors:** Radosław Wolniak, Wiesław Wes Grebski

**Affiliations:** 1 Department of Economics and Informatics, Faculty of Organization and Management, Silesian University of Technology, 44-100 Gliwice, Poland; 2 Engineering Faculty, Penn State Hazleton, Pennsylvania State University, 76 University Drive, Hazleton, PA 18202-8025, USA; wxg3@psu.edu

**Keywords:** food safety, RASFF notifications, pesticide MRL exceedances, food waste, regulatory compliance, food governance, sustainability initiatives

## Abstract

Guaranteeing food safety in the European Union (EU) is a continuing issue affected by diverse national traditions, regulatory power, and consumer culture. Despite the presence of a harmonized regulatory context, there continues to be variability in performance among the 27 member states. This study gives an extensive comparative evaluation of EU food safety based on three indicators: Rapid Alert System for Food and Feed (RASFF) alerts, pesticide maximum-residue-limit (MRL) violation, and per capita food loss. Fuzzy TOPSIS, K-means clustering, and scenario-based sensitivity tests are used to give an extensive appraisal of the performance of member states. Alarming differences are quoted as findings of significance. The highest number of RASFF notifications (212) and percentage of pesticide MRL non-compliance (1.5%) were reported in 2022 by Bulgaria, whereas the lowest values were reported by Estonia and Lithuania—15–20 RASFF notifications and less than 0.6% MRL violation rates. A statistically significant correlation (*r* = 0.72, *p* < 0.001) between pesticide MRL violation and food safety warnings was confirmed in favor of pesticide regulation as the optimal predictor of food safety warnings. On the other hand, food loss did not significantly affect safety measures but indicated very high variation (from 76 kg/capita per year in Croatia to 142 kg/capita per year in Greece). These findings suggest that while food loss remains an environmental problem, pesticide control is more central to the protection of food safety. Targeted policy is what the research necessitates: intervention and stricter enforcement in low-income countries, and diffusion of best practice from successful states. The composite approach adds to EU food safety policy discourse through the combination of performance indicators and targeted regulatory emphasis.

## 1. Introduction

Food safety is a pillar of public health and economic integrity in the European Union [1,2,3]. In an extremely dynamic and interconnected system of food, with diverse cultural practices and disparate socio-economic progression levels, food safety is a multifaceted issue that requires advanced solutions and sound regulatory systems [4,5,6]. This section offers the complete theoretical framework of the main issues that are the foundation of this study: the pillars of food safety governance in the European Union, the role of control and monitoring systems of pesticide residue, the issue of food losses, and the need for integrated data-based analysis in developing targeted policy.

Food safety encompasses all the practices and food chains that are concerned with averting foodborne illnesses and making the food safe for human consumption [7,8,9]. The World Health Organization (WHO) uses food safety as a main public health activity that guards individuals from health risks due to biological, chemical, or physical contaminants. Food safety is also important in terms of consumer confidence, economic productivity, and international trade [10,11,12].

This research bridges an essential gap in recent European Union literature on food safety [13,14,15,16,17,18,19] to the point of being founded on a quantitative and qualitative comparative analysis of all 27 EU members based on several key indicators. Whereas previous research has been more inclined to study singular components of food safety separately, for example, pesticide use or food loss, this work unites all of them in an organizing framework that can allow consideration of the way in which they interconnect with each other to generate national food safety landscapes [20,21,22,23,24]. With the employment of standardized information and using complex statistical methods, this article answers a genuine request for one method which is sensitive to the multidimensionality and complexity of the EU food safety issues.

One of the key indicators applied in this research is the maximum residue limit (MRL) of pesticides, that is, the upper limit of pesticide residue allowed in food or feed commodities in the European Union. Threshold levels are established pursuant to Regulation (EC) No. 396/2005 and aim to safeguard consumers against chronic toxicological effects of chemical exposure to farming foods. Scientific studies confirm that excess of MRLs would pose severe threats to human health, such as carcinogenic, endocrine, and neurological impacts [25,26,27,28,29]. Checking for MRL compliance is one of the mainstays of the EU system of food safety and is constantly carried out by the European Food Safety Authority (EFSA) by means of annual reports [30,31,32]. Exceedance rates can differ at the national level due to variation in agricultural practices, intensity of enforcement, and technological ability of food control laboratories [30,31,32,33].

It also includes the critical dimension that has been taken into account in the analysis, i.e., food waste, traditionally defined in terms of environmental and economic sustainability. Food waste has been shown to impose resource inefficiency, produce greenhouse gas emissions, and compromises food security goals [34,35,36]. More recent works have begun examining its indirect connection with food safety, i.e., where poor storage or handling of surplus food increases the probability of contamination [37,38,39,40]. EU policy reports like the Circular Economy Action Plan and the Farm to Fork Strategy explain that reducing food waste should be an integral part of a holistic approach to sustainability transitions toward food systems [41,42,43,44]. While wastage of food is not defined as a formal association with food safety notification volume, it may be used as a surrogate marker for systemic inefficacy and consumer practice concerned with the overall integrity and resilience of the food chain [45,46,47].

This study uses the Fuzzy TOPSIS approach and K-means cluster analysis innovatively to cluster and rank countries based on their performance on food safety measures and thereby portray more vivid pictures of national diversity than neat descriptive statistics. Our approach enables a long-desired reaction to a fundamental shortcoming of the literature, since subtle country-level distinctions and rankings have been the norm rather than the exception. The use of fuzzy logic, for example, offers a way of representing uncertainty and partial membership, more richly involving the variable, dynamic nature of the real world not necessarily defined by linear models. It allows policy makers to aim and prioritize interventions more precisely on the basis of enhanced conceptualization of systemic risks and resilience.

Through the use of scenario-based sensitivity analysis, this study fills another gap in existing research—investigating how policy-relevant variation in conditions like pesticide exceedance and food loss could impact the general food safety climate. Existing research in this field has lacked this forward-looking aspect in the majority of cases, making its applicability toward anticipatory planning of policies less desirable. The forward-looking methodology of this research findings can be used to better-informed decision-making, facilitating the development of strong and flexible food safety policy at both national and EU levels. This article presents an integrated and solid analysis that constitutes an enriched contribution to the European food safety governance debate.

In this research, three research questions were formulated:

• How do the 27 EU member states compare in terms of food safety performance based on key indicators such as RASFF (Rapid Alert System for Food and Feed) notifications, pesticide MRL exceedances, and food waste per capita?
• What are the main drivers behind differences in food safety indicators across EU countries?
• How can the findings inform future food safety policies and targeted interventions at the EU and national levels?

The main scientific contribution of this paper is the comprehensive, integrative performance analysis of food safety in all EU member states using a strong combination of sophisticated statistical methods—i.e., Fuzzy TOPSIS, K-means clustering, and scenario-based sensitivity analysis—to detect faint patterns and interdependences among the most significant indicators: RASFF notifications, pesticide MRL exceedances, and per capita food waste. By uniting these traditionally distinct dimensions under a single framework, this study provides a more developed, evidence-based explanation of food safety inequality and determines areas of priority for targeted policy intervention. Not only does this new approach contribute to the academic debate, but it also provides a pragmatic toolkit for policy makers who wish to develop successful, country-specific policy responses for food safety regulation in the European Union.

The theoretic value of this research lies in the holistic analysis framework where three unrelated but interrelated dimensions of food system performance—RASFF notices, pesticide maximum-residue-level (MRL) transgressions, and per capita food losses—are integrated under a single measuring framework. Standing alone from previous research using severed indicators or strictly descriptive approaches [20,21,22,23,24,25,26,27,28,29,34,35,36], the present research uses a multidimensional measure with the application of State-of-the-Art techniques such as Fuzzy TOPSIS, K-means clustering, and scenario-based sensitivity analysis to unveil hidden interrelations and structural variations among EU countries. It does this by not only building the ideational pillars of food safety regulation within the EU setting but also resisting the application of the usual dichotomy between the regulation of safety and sustainability indicators. The research indicates that food policy must address the regulation implementation, agricultural practices, and consumer attitude all at once in its pursuit of both safety and system resilience.

## 2. Theoretical Background

The European Union has long acknowledged the primacy of food safety over sustainable development and public health. By building coherent regulatory frameworks—like the General Food Law (Regulation (EC) No 178/2002) [48]—the EU has developed a framework that provides a high level of protection throughout member states. Interestingly, variations among countries still remain, which can be traced back to history, culture, and structural variations in food production and monitoring systems [49,50,51].

One of the EU’s greatest success stories in food safety regulation is the establishment of the Rapid Alert System for Food and Feed (RASFF). Beginning in 1979 and updated from time to time, the RASFF system offers a framework for exchanging information regarding direct or indirect hazards to food safety among member states in real time [52,53]. The system allows for rapid response to food issues—product recall or public warning—averting possible harm to consumers [54,55].

The number of RASFF notifications made available by individual member states has emerged as a commonly used indicator of food safety performance. Recurring high rates of notification can indicate active vigilance and assertive risk identification, while recurring low rates can indicate good prevention work or, alternatively, poor monitoring [56,57]. Such indicators therefore need to be interpreted in the context of a sophisticated understanding of each country’s regime of regulation, as well as transparency practices [58,59,60,61,62,63,64,65].

Pesticides, though important in current agricultural production, are a food safety issue because of the potential effects on health when residues in foodstuffs exceed regulatory levels [25,26,27,28,29]. The EU has established strict maximum residue levels (MRLs) for pesticide residues in food and feeding stuff under Regulation (EC) No. 396/2005 [33]. Compliance with such MRLs is an integral aspect of food safety law intended to safeguard consumers from long-term health threats posed by pesticide residues.

Scientific studies have proven that exposure to pesticide residues through diet can cause an array of diseases, from neurotoxicity and endocrine disruption to carcinogenicity. EFSA has the most critical function in risk assessment and data gathering in its yearly reports identifying the status of pesticide residues in member states. Country rate differentials of MRL exceeding rates could potentially mirror differentials of farming practices, enforcement, and monitoring laboratory modernization extent [30,31,32].

Food loss has traditionally been studied primarily as an environmental and economic issue because of its value in the realization of resource efficiency, greenhouse gas emission reduction, and food security enhancement [34,35,36]. Recent perspectives recognize that food waste also has close associations with food safety. For instance, poor handling and storage of excess food can increase the risk of contamination, while unnecessary wastage can signal supply chain inefficiencies, thus lowering the standard of food safety [37,38,39,40].

The EU policy environment has increasingly acknowledged the value of addressing food waste, as expressed in the Circular Economy Action Plan and the Farm to Fork Strategy [41,42,43,44]. Nevertheless, numerous nations still record high per capita food waste, reflecting consumer behavior and system-level failures. This research incorporates food waste indicators into the framework analysis and supports an integrated view of food system sustainability and risk [45,46,47].

EU nations’ heterogeneity of food safety performance is the outcome of a multidimensional intersection of historical, economic, and socio-cultural determinants. Northern and Western European nations like Finland, Sweden, and the Netherlands have traditionally exhibited high regulatory control orientation and devoted significant resources to the construction of good food safety surveillance systems [66,67,68,69]. This governmental emphasis is supplemented with high consumer engagement and awareness that help in meeting stringent food safety regulations [70,71,72]. It boasts a very low pesticide maximum-residue-level (MRL) exceedance and an active strategy toward food waste management, reflecting both environmental consideration and public health concerns [73,74,75,76,77,78].

Southern and Eastern European nations tend to have structural flaws and institutions limiting their ability to achieve the same level of standards [79,80,81,82,83]. These are structural issues that include economic limitations to modernizing laboratory infrastructure and regulatory regimes, therefore creating gaps in the enforcement and differential application of EU-wide food safety policy [77,78,84,85,86]. In nations like Bulgaria and Greece, for instance, chronic rates of MRL excesses and excessive food losses can be indicators of such deeper structural problems [77,78,79]. The cross-interplay of stringent budgets, low institutional capacity, and dependence on traditional farming methods leads to the formation of a food safety environment that is more decentralized and dissimilar [80,87,88,89,90].

Apart from the economic and regulatory aspects, cultural attitudes related to food consumption and production are also pivotal in determining food safety outcomes [91,92]. There has been a vibrant cultural history of public criticism and consumer protest of food-making practices in most Northern and Western European nations. Such cultural attitude brings about a culture where one can expect the regulatory institutions to be highly transparent and accountable [93,94]. Southern and Eastern European countries might encounter cultural obstacles to the adoption of food safety best practice, e.g., lower rates of consumer engagement or resistance to the value of modernization. These cultural subtleties are difficult to overcome with policy tools alone, thus underscoring the imperative to apply specific education and communication measures that are congruent with national beliefs and values [95,96].

Together, these determinants paint a rich portrait of food safety performance in the EU. They indicate that differences in food safety activities are not simply the result of technical failure, but are rather the result of intersecting historical inheritances, economic contexts, and cultural inclinations. Confronting these differences therefore requires an equally rich approach: one that combines regulatory modernization with specialized capacity-building schemes and culturally sensitive consumer education. It is only by transgressing these various domains that policy makers may hope to construct an actually harmonized food safety environment that preserves the European Union’s high standard but is responsive to the various contexts of its member states [97,98].

They suggest the requirement for country-specific policy and not generic strategies. They suggest the value of taking a comparative approach which can determine both best practices and where targeted intervention is needed within the EU’s overall regime of food safety governance.

Current food safety research more commonly employs more sophisticated statistical tools to provide results that extend beyond mere descriptive analysis [75,76,77,78,79,80,81,82,83,84,85]. Researchers are enabled by multivariate analyses—clustering analysis and multiple linear regression—to uncover patterns and relationships that are unexpressed in univariate analyses. The techniques are beneficial to the formulation of evidence-based policies with a greater likelihood of providing concrete changes in food safety results.

Fuzzy logic techniques, for example, the Fuzzy TOPSIS technique employed here, are well suited to conditions under uncertainty and partial data. Linear models of conventional form can fail to realize the richness of food safety systems, where nations can simultaneously exhibit strengths and weaknesses. Fuzzy techniques allow for gradations of performance and supply a more realistic, as well as more informative, representation of relative food safety performance across nations.

While the available literature provides useful information for stand alone individual aspects of food safety—such as pesticide residues or food loss—there is little research that has synthesized these variables in a unified, coherent analysis that includes surveillance system efficiency through RASFF notifications. Such a gap denies policy makers and researchers the right to gain insight into the dynamic interlacings between these dimensions and the ability to synthesize global interventions that tackle causes rather than symptoms.

This report aims to bridge this gap by integrating RASFF notice-mentioned statistics, MRL exceedance records, and per capita consumer food waste into an integrated analytical framework. This framework recognizes that agricultural production regimes, regulatory system, and consumer behavior are interlinked in determining national food safety profiles. It enables cross-country comparison not just on absolute performance but relative strengths and weaknesses as well, offering a platform for knowledge sharing between countries and targeted policy assistance.

## 3. Materials and Methods

This study utilizes a quantitative large-scale approach for the comparative analysis of the food safety of 27 EU countries during 2022. This evaluation employs three leading indicators: total RASFF notices, percentage of maximum pesticide residue limit (MRL) exceedance rate, and food wastage per capita. The remedy for sources of data, preprocessing of data, and methodology adopted in this study is presented below.

The years 2022 and 2020 were chosen to capture the most recent states of food safety in the EU alongside a comparable pre- and peri-pandemic baseline. Using these two time points enables the identification of both short-term dynamics and persistent structural patterns across member states. Data for both years were fully available and standardized across all three indicators—RASFF notifications, pesticide MRL exceedances, and food waste—ensuring methodological consistency and reliability of comparative analysis.

The primary information collected in the research was obtained from trusted sources and official announcements from the food safety-regulating agencies. The notification information in RASFF was obtained from the RASFF report, which is considered to be one of the most useful sources to monitor the quality and safety of food in the EU. MRL exceedance statistics were collected from European Food Safety Authority (EFSA) reports, and food losses were collected by Eurostat and the statistical offices of member states.

Structurally and seasonally adjusted food safety extent was used for the purposes of comparability, and the data used were made public in 2022.

Reliability of the sources can be ascertained due to the strict data-collection and -filtering procedures used by EU institutions.

The data were treated with absolute regard for quality and comparison before any analysis.

Quantitative data on RASFF notifications, MRL violation of pesticide, and loss of food per capita were normalized by the min–max normalization method that converts values of the same range and minimizes variation in units.

Checks on quality were conducted to identify and correct missing or outlier values, which would cause bias while performing later analysis. Values imputed using national or regional means were used in the case of missing values if the case data lacked them so that the integrity of datasets could be maintained.

The research was conducted in several steps and utilized an array of statistical techniques and multivariate analytical methods, hoping that they would capture food safety performance complexities.

The initial work in the analysis was to perform descriptive statistics for all the indicators of each country, including mean, standard deviation, and value range. This made it possible for countries whose values are very low or very high for all the indicators to be identified at an early point and to receive structural data overview. This was performed through Microsoft Excel.

To obtain a compound estimate of food safety in the EU, Fuzzy TOPSIS was applied. Due to its ability to handle uncertainty and fuzziness in multi-criteria decision-making problems with high preference, the method yielded rich ranking of countries by similarity to an ideal level of food safety.

The computational procedure included the following:

• Establishing the decision matrix based on the three indicators.
• Normalizing the data using linear functions to ensure comparability.
• Calculating weighted values that reflected the relative importance of each criterion.
• Identifying the ideal and anti-ideal solutions to serve as benchmarks.
• Calculating the distance of each country from these benchmarks.
• Computing the closeness coefficient for each country, forming the basis for final rankings.

Cluster analysis was conducted to group EU nations based on food safety performance congruence. K-means was used because it is efficient and able to uncover clusters (groups) with the highest internal cohesion. Clustering entailed the following:

• Determination of the best number of clusters using the elbow method and plotting within-cluster sum of squares.
• Group-by-group assignment of countries with the objective of minimizing within-group variance.
• Determination of the clustering result, graphing similarity among clusters and areas of potential targeted policy action.

For revealing relations of magnitude and direction among the three indicators, Pearson correlation analysis was employed. This aspect helped shed light on possible structural relations and provided us with some insight into the relations that are worthy of using when subsequently informing public policy formulation.

Besides this, multiple linear regression was used to analyze further determinants of RASFF notification quantities. Pesticide MRL exceedance frequencies and per capita food loss were used as two independent variables in the model. Regression coefficients, *t*-values, *p*-values, and confidence intervals were derived to show the strength and significance of the associations. The results gave rise to a more refined account of how these variables are responsible for variation in notices to food safety and gave guidance to possible policy interventions.

As a test for sensitivity, sensitivity analysis was carried out to look at the impacts of percentage changes in explanatory variables on the volume of RASFF notifications. Semi-elasticities of all explanatory variables were estimated to find the size of these effects. Scenario tests were also run, estimating the impact of ±10% explanatory variable changes on estimated volumes of notifications. Scenario tests are a useful vehicle for projection of the consequences of hypothetical policy action.

The choice of Fuzzy TOPSIS, K-means clustering, Pearson correlation analysis, and multiple linear regression here was motivated by the requirements to combine multidimensional data, eliminate structural patterns, and measure statistical correlations in an open and legible way. Fuzzy TOPSIS was selected because it possesses the capability to accept imprecise and subjective opinions in a multi-criteria decision-making (MCDM) environment, making itself appropriate for composite food safety evaluation in uncertain situations. K-means clustering allowed for the separation of nations into comparable clusters based on their food safety and sustainability profiles and therefore revealing hidden typologies that could remain hidden within aggregate analysis. Pearson correlation was then applied to initially investigate the linear strength and direction of relationships between the more important variables. Lastly, multiple linear regression was used as a well-established traditional method of estimating the net impacts of chosen predictors—i.e., pesticide MRL exceedances and food losses—on RASFF notifications. All of these methodologies in turn represent a complementary and strong toolset for revealing both structural isomorphisms and causal linkages in European food safety dynamics, and they are methodologically simple and reproducible.

The Fuzzy TOPSIS model’s development was based on a multi-criteria decision process that followed a systematic approach suitable for the food safety and sustainability assessment situation. The decision matrix was constructed in the first step from normalized values of selected indicators applied to EU member countries. Triangular fuzzy numbers (TFNs) were utilized to resolve uncertain or judgmental weights, which were assigned to every criterion depending on preference scales based on experts from the literature and institutional risk-priority systems. The normalized fuzzy matrix was applied to calculate the fuzzy positive ideal solution (FPIS) and fuzzy negative ideal solution (FNIS). Euclidean distances between each option and the FPIS and FNIS were computed, and each country’s closeness coefficient was then established to establish a final ranking of food system performance.

For K-means clustering, the input parameters were all normalized prior to applying z-score normalization so that values from different scales could be compared. The number of clusters was decided using the elbow method, where WCSS is plotted against k and a point of diminishing returns is determined. Based on the inflection point of the elbow plot, a k value of 3 was chosen. The algorithm was executed under random initialization with reproducible fixed-seed guarantee and 10 iterations to ensure convergence. Cluster labels generated enabled the identification of spatial and structure patterns among member states, uncovering groupings with comparable pesticide infringements, food losses, and RASFF notification profiles.

The multiple linear regression models were constructed in order to estimate the combined impact of two significant predictors—pesticide MRL exceedances and per capita food loss—on the quantity of food safety warnings, as reflected through RASFF notifications. Ordinary least squares (OLS) estimation was used in the estimation of distinct models for each year (2020 and 2022). Before model estimation, diagnostic tests were performed to test multicollinearity (variance inflation factor, VIF), normality of residuals (by Q-Q plots), and heteroskedasticity (by Breusch–Pagan test). Apart from the full models with both predictors, reduced models including only MRL exceedances were also estimated in order to test the robustness and interpretability of causal inference. Model fit was tested by R-squared, F-statistics, and *p*-values of individual coefficients. After-the-fact sensitivity analysis was employed to measure RASFF response’s semi-elasticity with respect to MRL exceedance-level changes.

All computation and statistical analysis were performed with R 4.3.2 (along with respective advanced libraries for multivariate statistics and regression modeling) and Python 3.11.8 (with NumPy 1.26.4, pandas 2.2.2, and scikit-learn 1.5.0 libraries). Tool usage guaranteed reproducibility and accuracy and potential replication in the future in other similar studies.

## 4. Results

Table 1 outlines the summary of food safety indicators in 2022 across the European Union’s 27 nations, such as the number of RASFF notifications [99], percentage pesticide maximum-residue-limit (MRL) violations [100], and per capita food waste average [101]. Table 2 presents these data for 2020 (some of the data are collected every two years). These statistics offer a comparison of food safety performance, indicating considerable variation across EU member states. Although there are some nations with high RASFF alert or pesticide exceedance levels, there are others with comparatively low food waste levels, suggesting both structural and behavioral diversity in food safety and sustainability practices within Europe. Such a comparison dataset provides a basis for additional multivariate analyses and policy development in the area of food governance and public health protection.

The table data reveal wide variations in EU Member State food safety performance. Greece and Bulgaria, for instance, report high rates of pesticide maximum-residue-limit (MRL) exceedances of 1.3% and 1.5%, respectively, an indicator of potential lacunae in agricultural practice or regulation enforcement. Finland and Luxembourg report the lowest rates of MRL exceedances of only 0.4%, an indicator of improved pesticide management or rigorous monitoring processes in these states.

RASFF alert numbers are very high for states, from 212, the highest, for Bulgaria to a low of only 10 for Luxembourg. They could both reflect how fully national food control systems are established and the reporting is open. It is also possible that the countries with high levels of notifications simply have better traceability and detection, and that this can be taken as a good indication of good food safety governance.

Food waste per capita ranges from 76 kg in Croatia to 142 kg in Greece, both reflecting consumer behavior and food system performance. Ireland and Denmark, for instance, enjoy relatively high living standards but also relatively high food waste levels (135 kg for each of them). This suggests that socio-economic factors alone cannot account for differences in food waste and management practices in the food chain; cultural norms also play an important role.

These steps emphasize the interactive complexity of farm production systems, food safety regulation, and consumer preferences to influence country-specific food safety profiles. Such perceived heterogeneity lends robustness to the arguments for country-specific interventions that go beyond the level of regulatory strictness to include public awareness and food chain organization. Additional research would benefit from supplementing such qualitative results with these quantitative recommendations, i.e., e.g., case studies or interviews with stakeholders, to better understand the determinants of European Union food safety performance.

Table 2 presents overall EU member states’ 2020 food safety indicators based on three variables: RASFF notifications, percentage of pesticide maximum-residue-limit (MRL) exceedances, and food waste per capita. At the level of RASFF notifications, there exists an exceptional imbalance among countries: France recorded the highest number of notifications (531); Malta (498) and Ireland (297) followed; and Luxembourg (4), Greece (28), and Croatia (16) recorded some of the lowest. Such imbalance may be able to pick up not just heterogeneity in food safety performance, but also heterogeneous institutional alertness, report systems, or degrees of control system maturity. At the same time, there were 0.4–1.5% pesticide MRL exceedances in Bulgaria (1.5%), Greece (1.3%), and Romania (1.3%), indicating possible loopholes in regulation or pesticide control. Finland, Luxembourg, and Ireland reported the lowest exceedance rates (0.4–0.5%), which could indicate a higher degree of compliance or better monitoring systems.

Per capita loss of food in 2020 presents a comparatively median figure for the EU countries, between 76 kg/person/year in Croatia and 142 kg in Greece. The maximum food loss rates were recorded in Southern and Western European countries, such as Greece (142 kg), Luxembourg (139 kg), and Finland (137 kg), perhaps attributed to overconsumption tendencies, food wastes in retailing, or consumer food habits in high-income environments. At the same time, lower amounts were noted in some countries, like Croatia (76 kg), Slovakia (89 kg), and Hungary (90 kg), which might reflect rather moderate consumption patterns or variations in waste measures. These figures do reflect food waste, which, although not directly related to food safety alerts, is the most salient element of supply chain management and sustainability that, on the grounds of socio-economic, cultural, and infrastructure conditions, varies considerably across the EU.

The straightforward comparison of the 2020 and 2022 RASFF notification quantities reveals significant declines in the majority of the EU member countries. For example, Austria fell from 267 reports in 2020 to a 45 in 2022; Ireland, from 297 to 25; Finland, from 220 to 35; and Malta, from 498 to a 12. Such drastic plummeting is perhaps reflective of enhanced food safety measures in the country, but it may be a sign of decreases in frequency checks, resource deployment, or post-COVID surveillance system failures. All of these observations suggest the need to place institutional and operational contexts into food safety data analysis.

While it is notable that RASFF notifications have been volatile, the pattern of pesticide maximum-residue-limit (MRL) exceedances has been highly consistent over the two-year span of our study in most countries. Bulgaria, for example, had a steady rate of 1.5%, Greece had a steady rate of 1.3%, and Finland had an incredibly low rate of 0.4%. Such tenacity may suggest that MRL excesses are less an issue of short-term policy response and more an issue of deeply institutionalized farm practice and systemic capacity for enforcement. Inability to make visible changes can also suggest regulatory lethargy or continued trouble in pesticide use and compliance monitoring.

The temporal stability was also strong for the per capita amounts of food waste among the members of the EU. They were the same or nearly so in 2020 and 2022. Germany, for example, was consistent at 130 kg/person/year, France at 132 kg, and Greece at a high rate of 142 kg. These stable trends indicate that food waste is not such a short-term policy issue to tackle and more deeply rooted due to supply chain design and consumer choice. The stabilization indicates that without sustained campaign effort to enlighten people, logistic innovation, and regulatory pressure, attempts to stem food waste are destined to fail.

## 5. Discussion

### 5.1. Fuzzy TOPSIS

The European Union nation ranking by food safety, calculated by using the Fuzzy TOPSIS approach, is presented in Table 2. It consists of three columns: “Country”, “Closeness”, and “Rank”. The “Closeness” column is the closeness coefficient of each nation to the ideal food safe profile, where the higher the value, the better the performance. The “Rank” column is the rank of each nation in the ranking, which can be utilized to determine the nations with better food safety practices.

Table 3 presents the Fuzzy TOPSIS ranking of EU countries against three major food safety indicators: RASFF notifications, pesticide MRL exceedance rates, and per capita food waste. Each country receives a composite performance score based on lower values representing improved overall performance in terms of food safety and sustainability. The table shows clear disparities between the member states, with Estonia, Lithuania, and Croatia at the highest level, and Bulgaria, Greece, and Spain at the lowest level.

The results of our ranking analysis indicate that Estonia, Lithuania, and Croatia are leading the way; thus, they have the best food safety indicators among the other EU member states. Specifically, Estonia’s leadership is most well-justified due to the notably low rate of notifications in the RASFF system, low occurrence of pesticide MRL exceedances, and low food losses. This is irrefutable proof that a collaborative approach to managing the food supply chain can lead to real gains in food safety performance.

Leader countries like Croatia and Lithuania also have high consistency across all three of the indicators under review. Lithuania stands out with a very low rate of food wastage, reflecting high commitment to pro-environmental and socially responsible practice. For Croatia, the low frequency of pesticide MRL exceedances may indicate successful enforcement of agricultural and food safety regulations.

Conversely, middle-level countries—Germany, Spain, and Poland—have a moderate number of indicators considered for assessment. Though they possess lower “Closeness” indexes, they indicate a fairly good level of food safety management. However, in these countries, additional specific public policies may be required, especially concerning the minimization of food loss, which is revealed to be among the key factors that reduce their overall indexes.

Ranking at the lowest are nations such as Greece and Bulgaria, which have much higher levels of pesticide MRL exceedance and RASFF notification. This would mean that food safety monitoring systems for these nations should be strengthened with increased controls and campaigns. Moreover, the high percentage of food waste in Greece indicates the necessity for interventions for altering consumption habits and improved supply chain management.

The ranking outcome reflects substantial disparity in the treatment of the food safety problems by the EU member states. Fuzzy TOPSIS methodology, considering the fuzzy interactions among indicators, provides a better insight into current differences and isolates nations that could be considered as a best practice model. Finally, these studies can serve as a foundation for policy suggestions to harmonize food safety policy across the European Union.

### 5.2. Fuzzy Clustering (Fuzzy C-Means)

Table 4 shows the comprehensive picture of clustering results of the European Union countries achieved by applying the K-means clustering technique. It includes data for every country, i.e., the cluster the country is included in; original values of three food safety indicators (RASFF notifications, percentage of pesticide MRL exceedances, and per capita food waste); and normalized indicator values. This overall picture allows us to compare food safety profiles differentially throughout the EU and to draw more specific conclusions regarding cluster differences.

Our analysis of the three-cluster results of EU nations along three dimensions—the number of RASFF notifications, pesticide MRL exceedance rate, and food wastage rate—identified stark variations in the approaches pursued by these countries to dealing with food safety and resource management. The application of the K-means technique, which enabled countries to be grouped into three clusters based on their characteristics, makes it easier to identify the common attributes, as well as areas for policy action. This exercise not only benefits researchers but also policy makers who are taking steps toward enhancing food safety and food system sustainability.

The first group contains countries with the highest number of RASFF notifications and exceedance of pesticide MRL. This group has a mean of around 91.7 notifications and 1.19% MRL exceedance on average. These are clearly higher than in the two remaining groups. The food waste indicator in this group averages around 125.5 kg per capita annually. This group suggests the necessity to improve food safety control and monitoring measures in these countries, along with public awareness to reduce food wastage. The internal heterogeneity of this group can possibly be due to differences in reporting measures and enforcement of regulation, but it definitely refers to issues that require a master plan of interventions.

The second cluster is also of interest, as it comprises nations which have the lowest values for all three of the indicators under review. The mean quantity of RASFF notifications here is as low as 28.2, and the mean MRL exceedance is just 0.82%. The percentage of food waste is also lowest among the clusters, at 89.5 kg a year per capita. These nations are considered best-practice models of food safety management. The performance reflects the presence of proper control systems for food quality and safety, aiming to reduce health and environmental hazards. It must be added that the low values of the indicators may be a consequence of high public awareness and cultural orientation toward good food management. Knowledge of the success factors of such nations can give lessons to others whose outcomes are less fortunate

The third group is nations taking a mid-level position—whose values are neither the highest nor the lowest. On average, the number of RASFF notifications for this group is approximately 34.2, and MRL exceedances stand at 0.53%. In contrast, in this group, the highest per capita quantity of food waste occurs, at 135.8 kg per year. This indicates that there is a particular profile for this group, with national control systems of food safety being extremely effective (low MRL exceedance and moderate RASFF notifications), but with a definite issue of food waste. This suggests that, in these nations, there can be a gap between food surveillance safety and the effectiveness of the whole food chain, whereby the losses are incurred at the stage of consumption or distribution.

In comparing the three clusters, it is clear that there is no straightforward linear relationship between RASFF notifications, MRL exceedances, and food waste levels. Though these indicators are conventionally associated, low-MRL-exceedance countries may have high food waste levels. This is an indication that more convergence of food safety activities with overall sustainability and consumer education activities is needed. The cluster analysis outcomes emphasize the importance of a comprehensive strategy incorporating both quality control and safety, as well as reducing waste, in order to address the entire supply chain.

The cluster analysis not only validates the EU member states’ grouping on food safety but also indicates avenues of special focus. The first-cluster countries require serious support in alleviating MRL exceedances and strengthening reporting mechanisms, whereas the second-cluster countries can be models of best practices. The third-cluster countries require efforts to minimize food waste. These findings offer a useful basis for the development of public policies, as well as for research—particularly in the context of climate change and increasing pressure on food systems in the European Union.

The European Union country-clustering results offer a useful corpus of knowledge for the development of effective and uniform public policies in the field of food safety. They help to identify those states which most critically require assistance in complying with sanitary norms, food quality controls, and consumer protection. Thus, policy makers can formulate policies according to the very needs of every state, unlike the “universal” approach, which fails due to European cultural and economic heterogeneity.

On the basis of EU country clustering within sector in food safety, it is possible to outline a comprehensive public policy package that addresses the diverse needs of each cluster of countries. The following is a specific multidimensional policy framework:

1. For the first-cluster countries (high frequency of RASFF notifications and MRL exceedances; moderate–high food losses), we make the following policy suggestions:
  • Improving control and monitoring systems.
  • Set up programs for food safety laboratory modernization and expand the number of food safety inspectors. Apply innovative digital technologies (e.g., tracking and traceability systems) to enhance the reporting of food safety cases.
  • Organizational and financial support.
  • Lend EU community funds or grants to provide programs allowing these nations to install best-practice-based and EU standards-based integrated food safety systems.
  • Education campaigns for producers and consumers.
  • Initiate agri-food producers’ programs to improve crop quality and minimize pesticide use, and consumer programs for responsible consumption and minimization of wastage of food.
2. For the second-cluster countries (low rating on all indicators; international high standard of food safety), we make the following policy suggestions:
  • Enhancing best practice at the European level.
  • Set up a “hub of knowledge” or “platform for dialogue” by which these countries can exchange experience and good practice with other EU countries.
  • Sustaining high levels of surveillance—enforcement of present policies, e.g., food quality monitoring, traceability schemes, and consumer education campaigns to preclude fall in performance.
  • Innovation incentives—encourage innovative projects to further minimize food waste, e.g., by creating technologies that enable food surplus recovery and redistribution.
3. For the third-cluster countries (low rates of RASFF and MRL, but high food waste), we make the following policy suggestions:
  - Programs for reducing food waste.
  - Implement national policies to minimize food loss at every phase of the food supply chain—production and distribution up to consumption.
  - Civil society and NGO involvement. Act in cooperation with other non-governmental actors engaged in food banks and information campaigns in order to encourage consumers to change their behavior and prevent food waste.
  - Increased use of data—establish national monitoring systems for food losses that can assess the effectiveness of implemented measures and coordinate policy adaptation to changing conditions.
4. Common EU-level actions include the following:
  - Funding cross-border activities of projects (e.g., university consortia and control institutions) in order to encourage innovative technological and systemic solutions.
  - Monitoring progress through regular comparative reports that indicate cluster-level performance and identifying areas of need for continued intervention.
  - One EU-wide campaign to put a focus on the significance of food safety and sustainable development in the European context.

The findings of this study demonstrate differences between some EU member states in their performance on food safety, as expressed in RASFF notifications, pesticide MRL exceedances, and per capita food waste. Estonia, Lithuania, and Croatia, which consistently topped the Fuzzy TOPSIS model and were part of the most preferred cluster, performed consistently well across all three indicators. This can be attributed to a mix of the following: comparatively recently and centrally organized food safety control administrations, close coupling with EU-level reporting systems, and a developing regulatory quality culture within the public administration. These nations are also most likely to be more willing to implement traceability technologies and facilitate transparency between producers and enforcement authorities.

Bulgaria, Greece, and, to a lesser degree, Spain repeatedly perform poorly on both high MRL exceedance levels and high RASFF notification levels. In these instances, deeply rooted structural obstacles would seem to be at work, such as under-resourced laboratory facilities, dispersed food safety enforcement, and insufficient institutional capacity to promote compliance. There are also likely to be cultural reasons—Southern European states especially have lower consumer involvement in food safety and more dependence on traditional farming practices that are not entirely suited to integrated pest management approaches. Finally, excessive food waste in nations like Greece is also an indication of inefficient consumption patterns or less efficient redistribution systems, which also encroach on efforts to move toward more general sustainability objectives.

For the supposedly “middle-tier” nations like Poland, Spain, Germany, and Portugal, there is a mixed performance profile. Poland, for example, has a medium degree of RASFF alerts and high MRL exceedances but comparatively low levels of food waste, indicating that while agricultural regulation might require tightening, post-harvest distribution systems are operating with medium efficiency. They need policy responses that are specifically designed for them since they have different risk profiles that may entail sectoral concerns. Refinement of agricultural methods with unhindered supply chain functionality could yield high dividends. Such sophistication warrants the necessity for multidimensional analytical frameworks that consider how institutional form, production practice, and consumer behavior interact to configure national food safety results.

### 5.3. Correlation Analysis

Table 5 is a correlation matrix of three major indicators of food safety in European Union nations (2022): notifications of RASFF, rates of exceedance of maximum residue levels for pesticide, and per capita rate of food waste. The data allow us to examine the direction and magnitude of interdependence of these variables, a point of departure for further causal and comparative investigation. Pearson correlation coefficient was used to calculate correlations, thus making it possible to determine linear relationships between the studied indicators.

Correlation matrix analysis reveals a highly positive and high correlation between the number of RASFF notifications and the percentage of pesticide maximum-residue-limit (MRL) exceedances. The Pearson correlation coefficient, which is about 0.72, indicates that countries with higher numbers of RASFF notifications also tend to have higher percentages of pesticide exceedances in their farm production. This is either because of common structural determinants, such as less stringent surveillance practices or less compliance with EU regulations in these countries.

It should be noted that the correlation between per capita food losses and the frequency of RASFF notifications is considerably weaker, with a correlation coefficient of about 0.20. This indicates that, despite the presence of some weak positive associations, it does not imply systematic or strong correlation between these two events. Equally, the almost-zero correlation of pesticide exceedances and wastage of food shows that the two problems exist independently of each other and rely on differing socio-economic and consumer-based mechanisms.

From the operational perspective, such results are of great importance for the design of public policy. The high correlation between pesticide exceedances and RASFF notifications for the countries where these phenomena are widespread implies the necessity of strengthening phytosanitary control and food quality assurance in those states. On the other hand, the loose connection between food safety markers and food waste indicates that the solutions to food waste necessitate independent initiatives—such as consumer behavior-change programs, improved supply chain logistics, and implementation of cutting-edge food waste-minimization technologies.

The debate also invokes the necessity of more sophisticated statistical models that also consider additional explanatory variables, e.g., levels of economic development, investment in food safety infrastructure, and cultural values. Regression models or cluster methods based on multiple variables would offer a finer grain of understanding of the mechanisms propelling the higher or lower indicator levels in countries. Such analyses would be of great utility to public decision-makers and global institutions interested in assisting member states with better food safety outcomes and sustainable supply chain management.

The correlation matrix for food safety indicators in EU countries in 2020 (Table 6) presents the relationships between three key variables: RASFF notifications, pesticide MRL exceedance rates, and per capita food waste. It shows a weak negative correlation between RASFF notifications and pesticide exceedances (−0.176), and a weak positive correlation between RASFF notifications and food waste (0.242). The correlation between pesticide exceedances and food waste is effectively zero (−0.001), indicating these factors operate independently in the 2020 dataset.

In the case of the 2020 percentage exceedance of pesticide MRLs and RASFF notifications, a weak negative correlation can be observed (*r* = −0.176). This means that rising levels of pesticides in exceedances never always indicated rising cases of food safety via the RASFF system. This is contrary to theory, as well as the 2022 snapshot, where correlation was very positive. This 2020 result could be explained by the diversity of national reporting regimes, differential levels of regulation compliance, or failing inspection schemes, perhaps resulting from the COVID-19 pandemic.

There is a moderate positive relationship (*r* = 0.242) between per capita food loss and food safety notifications via RASFF. This indicates that countries with higher rates of per capita food loss experienced a slightly higher rate of food safety notifications, but the relationship is not robust. The relationship can be explained by common root causes such as amounts of food consumption and production, inefficiency in the supply chain of food, or eating habits. Without the low correlation strength, though, it would not be wise to assign any causation until further research is performed.

The correlation between pesticide MRL exceedances and per capita food wastage is zero (*r* = −0.001), indicating that the two measures were statistically independent in 2020. This is in line with theoretical differentiation between pesticide-related food safety hazards—predominantly linked with agricultural practice and control—and food wastage, which is more tightly linked with consumer habits, retail supply chains, and cultural values related to food consumption. Keeping this in view, pesticide exceedance reduction policies and food loss reduction policies must be deployed through autonomous, expert mechanisms that are appropriate for their respective drivers and contexts.

Comparison of the correlation matrices of 2020 and 2022 reveals that there is a spectacular relationship change between pesticide MRL exceedances and RASFF notices. In 2020, the correlation between the two variables was weakly negative (*r* = −0.176), as there was no association and minimal relationship between pesticide regulation offenses and reported food safety issues. On the contrary, in 2022 the correlation was very high (*r* = 0.722) such that in the next year those states with higher pesticide exceedance would be much more likely to report food safety cases using the RASFF scheme. Alternatively, it is also a scenario where such a shift could be due to increased linking between the schemes, increased enforcement, or increased traceability and reporting capacity of states.

The association between RASFF notifications and food loss was higher in 2022 (0.200) than it was in 2020 (0.242), albeit still weak and statistically insignificant during both years. The near-zero association in 2020 (*r* = −0.001) between pesticide MRL violation and food loss for 2020 was also the case for 2022 (*r* = 0.003), once again affirming that the two measures are picking up on entirely different aspects of food system performance. Temporal comparison implies that while causality between pesticide offenses and alerts on food safety has drawn closer—possibly because of more responsive regulatory systems—food waste is a standalone issue of sustainability to a considerable extent, unaffected by food safety policy.

### 5.4. Multiple Linear Regression

Table 7 is a descriptive overview of the results of the multiple linear regression (2022). It contains coefficients, standard errors, t-values, *p*-values, and 95% confidence intervals for all parameters in the model: the intercept, the number of pesticide MRL exceedances, and the per capita rate of food waste. Such a detailed presentation allows it to be easily noticeable for each predictor what is its magnitude and statistical significance of the effect on the number of RASFF notifications in EU nations.

Multiple linear regression outcome delivers detailed analysis of the factors determining the number of notifications to RASFF made by the European Union member states. The predictor variables utilized in this outcome include percentage of exceedance of pesticide maximum residue limit (MRL) and per capita volume of food waste. The R-squared coefficient of determination was approximately 0.56, and this means that 56% of the variation in the number of notifications is explained by these two variables. This is an extremely robust result for socio-economic analysis, considering the reality that food safety in the real world is determined by a multitude of other factors not covered by this model.

Observation of the regression coefficients at closer range reveals the particularly significant role played by pesticide MRL exceedances. The coefficient of this factor is −94, and its *p*-value is significantly less than the 0.05 significance level (*p* < 0.001), which clearly suggests the significant influence of this factor on the number of notifications in the RASFF system. It indicates that there is also a significant increase in the number of food safety problems reported in the US, where the level of pesticide residues increasingly exceeds acceptable levels. This agrees with intuition: increased prevalence of elevated pesticide residues merely translates into increased health risk and increased warnings in the European warning system.

Food waste was not statistically significant in estimating the frequency with which RASFF notifications occur in the estimated model. Although the regression coefficient on food waste is positive (approximately 0.37), its *p*-value is 0.16, which is above the conventional level of 0.05. This would mean that, on this linear model and dataset, food waste is not a significant explanatory variable in variance explanation in the quantity of food safety notifications. Food waste is, however, an environmental and social issue of high visibility that is deserving of examination on its own merits, perhaps in comparison to other variables such as consumer attitudes, education, and supply chain logistics.

Interestingly, the intercept term of the model is statistically significant (*p* < 0.05), and one gets the feeling that there may be other impacting variables for which this research has not taken into account. Some of such variables may include administrative control action efficiency in each country, investment in emerging-generation food traceability technology, or public awareness level on food safety.

Such findings based on multiple regression are useful, as well as research-based. They provide solid case arguments for public policy makers and regulators to place the area of food safety at the top of their agendas. The significant and high impact of pesticide MRL exceedance is evidence of the need to maintain phytosanitary control by those countries having excessive pesticide residues, as well as to give training and consultancy services to farm producers. Stricter control and support for programs that decrease pesticide use would lead to a drastic reduction in food safety notifications and a general improvement in food quality.

It provides a foundation to construct more advanced analyses. For instance, it could be broadened to hierarchical or multilevel models that adjust for regional and national levels of variation within agricultural practice and food safety monitoring systems. Moreover, if one had data over a few years, panel data models could be used to examine the dynamics of these relations over time and determine long-term trends.

The results also have a significant role to play in public communication. They can serve as a statement of argument in information campaigns for alerting the public to the seriousness of the damage that results from the infringement of pesticide limits. Stressing the interdependence of agricultural production and consumers’ health can be a crucial component of an effective prevention policy and of creating a culture of responsibility throughout the entire food supply chain.

The 2020 multiple linear regression (Table 8) provides an idea of the inter-relationship between food safety notifications (RASFF) and two major predictors: pesticide MRL exceedances and per capita food waste. The model is well-fitted and has a high explanatory power, as can be seen by the fact that R-squared is high (not in the table, but taken for granted in the full study). The regression coefficients yield direct effects, and both independent variables are statistically significant in explaining the variation in the volume of RASFF notifications by EU members.

The model intercept is 135.429 and statistically significant (*p* = 0.008), meaning that even if pesticide MRL exceedances and food losses were equal to zero, the baseline notification level of RASFF would be above 135. This is evidence that there are some unquantified factors, i.e., institutions’ performance, traceability technologies, or trade intensity, affecting notification levels that should be included in future models.

Exceedance of the pesticide MRL is strongly negatively and statistically significantly correlated with −112.508 (*p* < 0.001), opposite to what was established in 2022, where the correlation was positive. This indicates that increased MRL exceedance in 2020 was associated with fewer RASFF notifications. One possible explanation could be initial underreporting or poor surveillance machinery during the pandemic, representing a decoupling between actual pesticide hazard and regulation. Alternatively, countries with poor reporting infrastructure could have high exceedances, as well as low notifications, in the form of poor enforcement and lack of transparency.

Per capita food waste was also discovered to have a statistically significant and negative relationship with RASFF notifications (coefficient = −0.773, *p* = 0.021). While the effect size is smaller, it does show that countries with higher food wastage experienced lower food safety warnings throughout 2020. This surprising result can reasonably be the result of complex system dynamics where food is wasted before safety issues are even identified, or it might be an indicator of less underreporting and reduced public awareness. It is also likely that some other food waste conceals concealed contaminations, particularly within and at retail levels where the waste is not reported via official routes of food safety.

The 2020 regression results demand a wise understanding of food safety indicators in relation to institutional, behavioral, and infrastructural differences in EU member states. Inversion of anticipated correspondence (at least in the pesticide MRL exceedance case) has the implication of separating the meaning of avoiding ignoring the bigger picture, i.e., disruption by COVID-19 pandemic, and variation in data collection regime and enforcement ability. The findings point to further, possibly longitudinal, research into food safety regulation tension and how reporting faithfulness can mislead people between states about actual exposure to risk.

The variation in the 2020 and 2022 multiple linear regression results indicates a significant reversal of the RASFF notifications–MRL exceedances relationship. While in 2020, the MRL exceedances’ regression coefficient was −112.508, indicating a negative and statistically significant relationship (*p* < 0.001), in 2022, the same coefficient reversed to +93.967, though statistically significant (*p* < 0.001). This first-of-its-kind change indicates that, in 2020, those countries with greater pesticide exceedances strangely observed fewer food safety alerts to RASFF, presumably due to reporting irregularities, institutional disturbances, or pandemic surveillance gaps. In contrast, by 2022, under post-pandemic regulatory circumstances restored to normal again, the anticipated positive trend did indeed appear—higher rates of MRL excess were now associated with higher rates of food safety notices, reflecting higher amounts of monitoring and agreement between actual risk and the conducted processes.

Food loss per capita has an equally inverted but more complex pattern. In 2020, food waste was statistically and negatively related to RASFF notifications (coefficient = −0.773, *p* = 0.021), suggesting greater food waste was found with fewer warnings—possibly because the hazardous food was rejected prior to being found. In 2022, the coefficient was statistically insignificant (*p* = 0.158) but positive (+0.369), suggesting food waste was not a good predictor of food safety notifications anymore. This is a decoupling signal between food safety inefficiencies and formal mechanisms for food loss, bearing witness that while food loss is a significant issue of sustainability, it is far from being a self-evident proxy measure of the efficiency of food safety systems. Overall, the above findings exemplify the dynamic, context-dependent nature of food safety regulation, and the need for adaptive, multi-factorial policy measures.

Table 9 and Table 10 present the estimates of basic linear regression models forecasting the relation between pesticide maximum-residue-level (MRL) exceedances and food safety alerts issued by the RASFF system during 2020 and 2022, respectively. Table 1 indicates that, during 2020, there was a statistically significant inverse relationship between MRL exceedances and RASFF notifications, and this could be evidence of underreporting or breakdowns of surveillance systems as a result of the COVID-19 pandemic. Table 2, however, indicates that, during 2022, there was a positive and extremely significant relationship whereby countries with greater pesticide exceedances were making significantly higher food safety notifications. This difference signals a change in the effectiveness of enforcement and sensitivity of regulation during these two time periods.

The linear regression models submitted here for 2020 and 2022 offer different information regarding the association between pesticide maximum-residue-level (MRL) exceedance and rate of RASFF (Rapid Alert System for Food and Feed) notifications among the EU member states. In both models, pesticide MRL exceedance served as the only predictor variable, allowing for a straightforward and targeted estimation of their impacts on reported food safety infringements. These findings are utilized to determine pesticide compliance as a distinct element of food safety regulation and introduce specificity by ruling out potentially misleading measurements of sustainability, like food waste.

The regression coefficient of exceedances in MRL was −112.51, and it was statistically significant at the 0.001 level in 2020. This paradoxical negative relationship suggests that countries with greater exceedance of pesticide residues strangely issued fewer food safety warnings. It is contrary to theoretical and policy logic, as well as a counterintuitive finding. One possible reason is the disruptions in systems brought about by the COVID-19 pandemic: decreased inspection capacity, diverted resources, and delayed reporting may have obscured the true food safety reality that year. In addition, those institutions with weak institutional arrangements can be expected to have underestimated pesticide violations and the resulting food safety risks.

The 2022 regression line demonstrates a closer theory-consistent and more cumulative relationship. The coefficient estimate of MRL excesses is +94.04, which is also extremely significant (*p* < 0.001), meaning an increase by 1 percentage point in MRL violations is associated with a 94 RASFF notification increase on average. This extreme positive correlation supports the evidence that pesticide abuse is one of the key causes of food safety risk in the EU. The rise in explained variance (R^2^ = 0.52) also supports the stability of the 2022 model, particularly since a single predictor was used. It can also represent heightened synchronization among detection, reporting, and regulatory response systems during the post-pandemic period.

The abrupt reversal in direction of association between the two years is an indication of changing institutional dynamics and not changes in underlying pesticide risks. Precisely, although the pesticide treatment patterns and cultivation practices were presumably quite consistent, the revival of inspection activities and surveillance systems in 2022 presumably enabled enhanced detection and reporting of infractions. This change indicates that enhancing food control infrastructure—instead of just reforming policy—is essential to ensuring alignment between real health risks and tangible regulatory deliveries.

The intercept coefficient of the 2022 model (−30.90, *p* = 0.082) was statistically on the margin, with the implication that, in the hypothetical situation where there were no MRL exceedances whatsoever, the predicted number of RASFF notifications would be practically zero. This gives weight to the pivotal role that pesticide residue violations have in initiating control warnings. Conversely, in 2020, the intercept was 135.43, indicating that, even without observed violations of MRL, food safety alerts would be noticed. This difference can account for the effect of the unobserved variables in 2020—microbiological or packaging risk, for example—or indicate over-reliance on RASFF data as too high an estimate of food safety in general.

The models highlight the importance of addressing food safety data institutionally and longitudinally. The 2022 findings affirm pesticide control as a pillar of food safety policy, and the 2020 findings prioritize the vulnerability of surveillance systems to crisis. Together, these findings underscore the importance of not only imposing stricter policies on pesticide control but also ongoing investment in monitoring capability, transparency, and institutional resilience. In employing a year-specific interpretation, the analysis resists over-hasty conclusions and more adequately informs strategic realignment of food safety interventions throughout the European Union.

Our full and trimmed regression model comparison for the years 2020 and 2022 reveals a clear disconnect in both explanatory congruence and statistical validity. In complete models, both pesticide MRL exceedances and food wastage per capita are employed as explanatory variables of RASFF notifications. Only the 2020 model provides statistically significant estimates for the two variables—but in the reverse direction. That is, both MRL exceedances and food wastage have negative coefficients, meaning higher infractions or higher waste are associated with lower food safety notifications. These findings, while statistically significant, probably represent distortions of the COVID-19 pandemic, i.e., disturbed inspection regimes or underreporting, and not real drops in safety conditions.

Conversely, the 2022 full model presents a very different scenario: pesticide MRL exceedance coefficient is positive and strongly significant, while food loss continues to be statistically insignificant. This implies a restoration of regulatory rationality, whereby the abuse of pesticides is a significant and quantifiable driver of food safety notices. Contrary to expectations, food loss—albeit environmentally and financially significant—is not a significant driver of RASFF notices, and its inclusion does little to enhance model fit. This underlines the argument against considering it to be a central concern of food safety research.

The reduced models, with pesticide MRL exceedance as the sole explanatory variable, yield the most interpretable and comparable results. For 2022, the reduced model performs almost as well as the full model, with a high R^2^ (0.521) and statistically significant, strong coefficient. In 2020, the smaller model also mimics the negative correlation which occurs in the larger model and verifies the argument that outcomes for that year are also likely to be influenced by institutional disruption. Combined, these results verify that pesticide MRL exceedances are the only strong and consistent food safety alert predictor in each of the two years, yet food waste—albeit of broader systemic concern—is best treated outside of main food safety mechanisms.

### 5.5. Sensitivity Analysis

Table 11 presents the sensitivity analysis of the multiple linear regression model in more detail for 2022. The coefficient, mean of the explanatory variables, and corresponding semi-elasticities are reported in the table. The sensitivity analysis shows each predictor’s one-percent change as captured by the change in the dependent variable, i.e., RASFF notifications. The conclusions are an informative source to determine which variable affects food safety occurrences in EU nations to the greatest extent, and thus which region is most likely to be most effectively targeted with policy intervention or action.

The sensitivity analysis of the multiple linear regression model tells us the relative effect of the two independent variables—pesticide MRL exceedances and food waste per capita—on the EU member states’ number of RASFF notifications. The semi-elasticity for pesticide MRL exceedances is also as high as about 105.27, revealing the significant contribution of this independent variable to the dependent variable. Conversely, that of per capita food loss is very low, approximately 0.0033. The suggestion is that the number of RASFF notifications is extremely sensitive to change in pesticide MRL exceedances but considerably less sensitive to change in food waste levels.

The size of the semi-elasticity of pesticide MRL exceedances also goes a long way toward emphasizing the key role played by agricultural practice and regulatory compliance in shaping food safety outcomes. Even a slight percentage rise in the number of MRL exceedances will result in a significant rise in RASFF notifications, emphasizing the need for strict regulation of the use of pesticides and more progressive agricultural practices. Nations that are prone to repeat MRL exceedance cases need to place utmost emphasis on policy reform in agriculture and invest in more effective monitoring networks to minimize such occurrences to the smallest extent.

The very low semi-elasticity of food waste indicates that the variable has no direct and significant impact on the quantity of food safety alerts. While food waste is a chronic social and environmental concern, it is to a great extent decoupled from the impending dangers that are caused by pesticide contamination of the food supply. This result is to be expected from previous regression results, which verify the hypothesis that food waste requires targeted policy interventions—like consumer awareness campaigns and supply chain efficiency measures—rather than intervention into food safety systems.

The overall conclusions of this sensitivity analysis offer policy makers and researchers clear directions. They validate pesticide MRL exceedances as the greatest cause of variability in food safety notifications throughout the EU, with food waste playing very little direct role. The response to minimizing food safety incidents should thus entail stricter regulation of pesticides, better practice in farming, and a shift to taking advantage of State-of-the-Art monitoring technology. Meanwhile, the development of a policy for reducing food waste needs to continue as a standalone priority among more general efforts at food system sustainability, supplementing and not substituting for fundamental food safety measures.

Sensitivity analysis of the multiple linear regression model again shows the percentage of pesticide maximum-residue-limit (MRL) exceedances as the most important causal determinant of volume of RASFF notifications in EU member states. The high semi-elasticity value reveals that even a small rise in MRL exceedances causes an appreciable rise in food safety-related notifications. The evidence clearly depicts the importance of greater phytosanitary measures and enhanced agricultural practices, since these contribute significantly toward reducing health risks and product quality improvements throughout Europe effectively.

Low per capita value of semi-elasticity of food waste is actually a verification that, as important as food waste is as a social and environmental issue, on its own, it does not directly influence the level of food safety notifications. Policy intervention aimed at reducing food waste should therefore be sought as an independent area of public policy, augmenting but not supplanting food safety intervention. The results of this analysis can accordingly be used as a basis for prioritizing interventions and formulating prudent evidence-based policy measures within the larger framework of the European food system.

The sensitivity analysis of 2020 regression model (Table 12) shows the contrasted role of pesticide MRL exceedance and per capita food waste in understanding the intensity of RASFF notification in EU member states. The semi-elasticity of pesticide MRL exceedance is about −100.42, which indicates that an increase in MRL exceedance by 1% is related to the reduction of about 100 units in the expectation of RASFF notification. This seeming paradoxical outcome is significant statistically and should represent anomalies in the reporting mechanisms or disruption of inspection activity—presumably as a result of the COVID-19 pandemic—more than actual successful food safety improvements. The negative coefficient signifies a negative correlation, where increased pesticide exceedance is paradoxically associated with fewer reported food safety transgressions.

Conversely, semi-elasticity for the case of per capita wastage of food is around −0.0068, a number that is so close to zero, it logs no impact on RASFF notifications, a result showing that, again, despite the assistance provided by this second variable, levels of food wastage did not, in 2020, add anything perceivable to food safety notifications.

The difference between 2020 and 2022 sensitivity analyses is a striking reversal of the relationship between pesticide MRL exceedances and the quantity of RASFF notifications. In 2020, a 1% increase in exceedance was associated with a 100-notification decrease, indicating an unusual and inverse relationship, perhaps due to systemic underreporting or disruption of inspections caused by the COVID-19 pandemic. Conversely, during 2022, the same 1% increase yielded over 105 notifications, as expected, suggesting a relearning relationship between pesticide infractions and food safety notifications in a post-pandemic regulatory framework. In each year, the influence of per capita food loss was once more negligible, with semi-elasticities around zero, confirming its being an issue of sustainability and not one of on-the-spot food safety concern. This variation emphasizes how institutional capacity and outside world conditions may significantly affect food safety systems’ vigilance and transparency in the European Union.

Sensitivity analysis of the truncated regression models (Table 13) wherein RASFF notification is better explained by pesticide MRL exceedance in isolation shows a dramatic contrast between 2022 and 2020. For 2020, the semi-elasticity is −87.10, which shows that a 1% increase in the rate of average MRL exceedance is worth an estimated fall of 87 RASFF notifications. This negative correlation runs counter to theoretical expectation and is likely the result of systemic deviance and institutional dislocations during the COVID-19 pandemic, such as delayed tests, weak enforcement, and underreporting. Consequently, the 2020 outcome is not seen as an accurate measure of real food safety dynamics but an artifact of abnormal circumstances.

On the other hand, the 2022 specification has a positive semi-elasticity of 105.36, implying that a 1% increase in the extent of MRL exceedance is equal to an average increase of 105 RASFF notifications. This result is theoretically sound and statistically significant, thus further buttressing the position of pesticide non-compliance as a key factor behind food safety notifications in the European Union. The magnitude of this sensitivity also points to the possible critical value of taking effective measures for good pesticide regulation and monitoring enforcement. These findings support the impression that MRL excess occurrence rates are more than statistically significant signals of food safety hazard, but also extremely elastic in their impact and, thus, a strategic leverage point for food policy intervention.

Sensitivity analysis comparison between the complete and reduced regression specifications shows that semi-elasticity measures for pesticide MRL excesses are still comparable across both groups of specifications, as would be expected from this powerful predictor. The semi-elasticity in 2022 is around 105.27 in the complete specification and 105.36 in the reduced one, proving that interpretational power be means of MRL violations is not reduced with the omission of food waste. Likewise, in 2020, both specs provide negative semi-elasticities of about −100.42 in the full model and −87.10 in the reduced model, confirming the contention that the anomalous direction results from institutional distortions rather than model misspecification. Hence, the reduced models provide a cleaner image of the underlying causal mechanism, and including food waste in the full models adds conceptual noise without further predictive gain.

Table 14 presents scenario analysis results, demonstrating how future RASFF notifications in EU member states would be impacted by hypothetical ±10% variations in the proportion of pesticide MRL exceedances and of the proportion of capita food waste. It presents base values and four different scenarios, which allow for easy understanding of how the model reacts to plausible variations in these determinants. Such scenario assessments assist in pinpointing the most important reasons for food safety incidents and in guiding policy development in accordance with those results.

RASFF notifications actually must be reported as integers since one notification corresponds to one reported occurrence of a food or feed safety problem, i.e., illegal-substance detection, microbiological contamination, or packaging hazard. There is no fraction or half notification. In any official EU report, e.g., presented by EFSA (European Food Safety Authority) or by DG SANTE, the quantity of RASFF notifications per year per member state is always reported in integers.

The result of the scenario analysis is that the RASFF notifications’ total estimate is most sensitive to pesticide MRL exceedances. A 10% increase in pesticide MRL exceedances leads to a high increase in expected notifications to around 61.42 from the baseline scenario of around 53.04. A 10% decrease in pesticide MRL exceedances leads to a high decrease in estimated notifications to as low as 44.65. These results are consistent with the results of the regression models above and strengthen the fact that pesticide control is at the core of food safety guarantee in the EU nations.

Variation in the levels of food waste, while within the scenario results, has a much more subdued effect on the expected number of RASFF notifications. An increase in per capita food waste by 10% leads to a slight increase in the expected notifications to around 57.21, and a reduction by 10% leads to a drop to around 48.86. This comparative insensitivity is to emphasize that, though reducing food waste is an environmental and social objective, it is not the identical kind of pressure on the food safety alert system as pesticide MRL exceedances.

These scenario results are of immediate benefit to researchers and policy makers. They emphasize that making the reduction of pesticide MRL exceedances a priority can achieve substantial decreases in food safety notifications throughout the EU. Policies focusing on the reduction of food waste, on the other hand, must continue to be a priority for overall sustainability but will have no immediate effect on food safety warnings. These scenario analyses therefore provide a practical guide to more focused and effective policy development and resource investment in the most determining factors.

The results of this scenario analysis make it clear that policy makers must act with determination to minimize pesticide MRL exceedances in the agricultural and food-producing sectors. There will need to be a reinforcement of monitoring systems, an improvement in the level and quality of checks, and the delivery of targeted support to farmers to use safer and more sustainable types of agriculture. These specific interventions should result in tangible enhancement of food safety performance, considerably lowering the amount of food safety notifications registered in the RASFF system.

In the meantime, this research recognizes minimizing food waste as a supporting but separate policy goal. Minimizing food waste, while an attractive component of general sustainability efforts, is not directly connected to food supply chain safety in the same manner that pest control is. Decision-makers can thus accommodate both priorities—reducing food waste and ensuring pesticide safety compliance—within an overall food system policy agenda in a way that each issue receives targeted interventions for optimal impact.

### 5.6. Practical Implications

This paper’s policy significance is high and of primary concern to regulators, policy makers, and EU agri-food industry actors. The finding that the main reason for RASFF notifications was pesticide MRL-exceedance implications indicates the need for a non-ambiguous and action-oriented priority goal for regulation. Multi-exceedance nations can be addressed with specific measures like enhanced surveillance of pesticide use, strengthening of laboratory analytical capabilities, and education of farmers on integrated pest management. These measures are proven to be effective in order to manage pesticide residue health risk, as well as food safety performance in general.

The more aggregated country groupings into clusters, based on food safety indicator convergence and divergence, offer a best chance for policy differentiation. Rather than applying undifferentiated, “one-size-fits-all” policies, policy makers can utilize the evidence to create interventions that target each country’s vulnerabilities and strengths. For instance, nations with a middle level of risk will need substantial support for system enhancement management, as well as supply chain integrity, whereas nations with a middle level can best be helped with more customized food wastage reduction activities.

Scenario-based sensitivity analysis enables policy makers to take a handy glance at the effect of slight variations in key variables—in this case, pesticide exceedances—on food safety results. Policy planning is done on the basis of such vision so that governments are able to allocate resources more effectively and invest where maximum mitigation of food safety incidence is achievable. The evidence-based and integrative focus of the research contributes to the academic literature and, equally important, positions practitioners well to have at their disposal a strong set of tools with which to construct stronger, safer, and better-governed food systems within the European Union.

For agriculture and public health ministries and public administrations, the findings of this study provide a clear message to accord priority to the monitoring and control of pesticide residues on food. In countries with frequent occurrences of MRL exceedance, like Bulgaria, Greece, and Spain, where repeated RASFF notifications are also being experienced, there is a need for increased inspections, better laboratory facilities, and regulatory action. The cluster analysis also allows for the distinction of countries with similar risk profiles to more easily craft regional cooperation programs and make better use of EU-level aid tools like agriculture or EFSA funding.

For the food sector, more specifically for food exporters in food quality-regulated high markets, the results provide a strategic instrument for the evaluation of competitive position in the EU. Producers in the high-RASFF alert countries or with high levels of pesticide-rule violation can expect higher trade impediments, especially to high-compliance economies like Germany, the Netherlands, or Scandinavian nations. On the other hand, producers in high-performing countries—Estonia, Lithuania, and Croatia—are in a position to use such ratings in their quality assurance program and brand differentiation. The outcome can also guide the application of voluntary certification schemes like GLOBALG.A.P., HACCP, or ISO 22000.

At consumer associations and public health lobby groups, the results specifically suggest the direction in which food contamination risk and regulatory failure likelihood is higher. Sizable numbers of RASFF notifications might indicate effective detection mechanisms alongside structural vulnerabilities in food production or import regulation. Conversely, insignificant rates of MRL overreach and food wastage reflect high public awareness and efficient public-education policy. The organizations may use the findings of this study as a platform for focused awareness campaigns, lobbying for strong national regulations, and formulating consumer-oriented food safety indexes to guide public choice.

### 5.7. Discussion with Literature

The analysis in this study has shown the percentage pesticide maximum-residue-limit (MRL) exceedance to be the main force behind the quantity of notifications in the European Union’s Rapid Alert System for Food and Feed (RASFF). The results of multiple linear regression and scenario-based sensitivity analysis validate the fact that it is not indispensable to account for considerable growth in MRL exceedances in order to achieve noteworthy growth in notifications. Even minor growth in MRL exceedances can cause noteworthy growth in notifications. This result corroborates previous estimates by the European Food Safety Authority (EFSA), which emphasized the health effect of pesticide residues in food products [25,26,27,28,29]. These findings complement more theoretical and abstract models that place pesticide management at the center of promoting food safety in the EU.

This current research indicates a novel approach by combining classical statistical methods with fuzzy logic-based methods, specifically the Fuzzy TOPSIS method. As highlighted in the current literature [75,76,77,78,79,80,81,82,83,84,85], such fuzzy logic methods are notably suited to depicting countries’ imperfect and nuanced memberships in various food safety performance profiles. This goes beyond basic linear analyses in enabling a more pragmatic and realistic comprehension of the intricacy of food safety governance in Europe. This kind of methodological development is an enrichment to food safety management discourse that can be deficient within normative models.

The outcome of this analysis also indicates that, although per capita food waste is an important environmental and social issue, it has nothing to do with pesticide safety notifications. The multiple linear regression analysis reports that food waste has an insignificant coefficient (*p* = 0.16), implying that food waste and pesticide safety are simply disparate topics. This result concurs with existing research that has been centered on food waste as an independent issue under the arena of sustainability systems [34,35,36]. Such policies reducing food waste need to therefore be put in place alongside, but not in place of, food safety-focused policies.

The theoretical foundation for this research resides in an appreciation of the diverse economic, historical, and cultural conditions driving food safety outcomes within EU member states [49,50,51,66,67,68,69]. This research is reminded of earlier findings that North and Western European nations, with established laboratory infrastructures and high consumer anxiety, have consistently reported reduced levels of MRL exceedance [70,71,72,93,94]. In comparison to this, Southern and Eastern European nations experience institutional and economic challenges to their ability to conduct food safety surveillance [79,80,81,82,83]. The contrast is one that indicates deeper structural and cultural fault lines that need to be addressed in any effective response policy.

Altogether, the results of this research are a valuable theoretical contribution because they bring three traditionally distinct fields—pesticide residue monitoring, RASFF notification, and food waste—under a common analytical framework. In doing so, not only do the authors validate current theory on the influence of pesticide management on food safety outcomes, but they also introduce a new hybrid analysis framework. This approach, which combines classic quantitative methods with fuzzy logic methods, offers fertile ground for extension. Such research can be based on this approach by incorporating longitudinal data and qualitative understanding, thus further clarifying the complex nature of food safety governance in the European Union.

Strong statistical and scenario-based evidence presented in this study confirms that exceedances of pesticide MRL are the most powerful predictor of RASFF notifications among the member states of the EU, which is in agreement with previous results of the European Food Safety Authority (EFSA) and the corresponding scientific literature [25,26,27,28,29,30,31,32]. The correlation is also consistent with toxicological experiments showing the chronic health hazards involved in pesticide exposure, such as neurotoxic, endocrine, and carcinogenic effects. The result reinforces the case for increased phytosanitary control, and indeed more specifically in countries such as Bulgaria, Greece, and Spain, whose rates of MRL exceedance continue to be high. The results reinforce the argument that food safety regulation in the EU cannot be separated from agriculture regulation and that sound risk avoidance will involve investing in integrated pest management systems, data-driven surveillance methods, and in high-resolution pesticide laboratory capability.

Although food waste is most often brought under the broad policy umbrella of circular economy and sustainability policy [34,35,36,41,42,43,44], the findings of this research offer empirical support for its theoretical and practical distinction from food safety regulation. Both correlation analysis and regression analysis concur that per capita food waste does not have a significant effect on the number of RASFF notifications, with coefficients ranging approximately around zero [45,46,47]. These results resonate with new theoretical suggestions implying that food loss reduction, as critical for the mitigation of climate change and resource efficiency, does not necessarily optimize microbial or chemical safety outcomes. Food safety and food waste should therefore be addressed on the grounds of the complementary but different policy streams, the first stemming from the risk avoidance and the second from the behavioral economics, logistics, and waste valorization technologies.

This research adds to the increasing body of evidence supporting data-informed food governance by merging cutting-edge statistical modeling with scenario-based sensitivity analysis [25,48,75,76,77,78,79,80,81,82,83,84,85]. Using Fuzzy TOPSIS, K-means cluster analysis, and regression diagnostics, the research shows how reductions in one important parameter—e.g., a 10% increase in pesticide exceedances—can rapidly boost the projected number of anticipated food safety warnings. This responsiveness reinforces the necessity for anticipatory regulation, where policy choice is driven by forecast analytics instead of payback mechanisms. The research invokes existing EU food policy discourse that a transformation from static indicators to dynamic, active risk management systems, which are able to visualize and respond to potential threats, is a necessity [52,53,54,55,85].

The cross-country differences pinpointed in this analysis also mirror the general institutional and cultural food safety drivers. As emerged from earlier studies, the Northern and Western European nations—Finland, Sweden, and the Netherlands—invest more in monitoring infrastructure and have higher consumer involvement and trust in the regulating authorities [66,67,68,69,70,71,72,93,94]. Conversely, Southern and Eastern European nations are subject to structural impediments such as institutional capacity constraints, budget limitations, and resistance to regulatory modernization [77,78,79,80,81,82,83]. These findings are consistent with theories that emphasize historical legacies, public attitudes, and regulatory culture determining national food safety performance. The implications are evident: EU food safety harmonization cannot be based on the adoption of standardized legal instruments but must be backed by support strategies with variability based on institutional maturity and cultural sensitivity.

Finally, the research adds to methodological contributions in food safety evaluation through the application of fuzzy logic-based methodologies—specifically Fuzzy TOPSIS—coupled with traditional statistical approaches [75,76,77,78,79,80,81,82,83,84,85]. This hybrid method permits the expression of uncertainty, continua of performance, and partial membership in risk categories—a further fruitful extension beyond binary or threshold methods. Through integration of three dimensions—pesticide residues, food alerts, and food waste—within one analytical framework, the investigation breaks through disciplinary boundaries and creates a more sophisticated understanding of systemic vulnerabilities. In doing so, it fills a central gap in the literature: one of the holistic models that bring together the multidimensionality of food safety and sustainability in the EU environment. The framework can be applied as a reproducible instrument to comparative analysis in the future, encompassing temporal dynamics, qualitative analysis, and regional clustering of food policy systems [75,76,77,78,79,80,81,82,83,84,85,97,98].

The transnational differences in food safety performance within the European Union can be best accounted for using the framework of institutional theory [102,103,104,105], highlighting the significance of formal structures, regulatory standards, and normative expectations in creating organizational and systemic results [106,107]. In this view, variations between food safety indicators—like RASFF notifications or MRL exceedances—are not technical outcomes but rather indicate national systems of food governance’s institutional maturity, legitimacy, and capacity. Those nations which score consistently high, such as Estonia and Lithuania, appear to benefit from institutional arrangements of isomorphism through aligning their national food safety systems closely with EU standards and norms and thus with consolidating both their legitimacy in the European policy space and their functional effectiveness.

Poorer performance in Greece and Bulgaria may be due to institutional decoupling, whereby there is symbolic adherence to EU standards in law but not necessarily internalized within everyday practice or enforcement mechanisms. Symbolic–practical disconnection typically is the result of a combination of weak administrative capacity, dispersed regulatory agencies, and less secure public trust in formal institutions [108,109,110]. Historical–institutional path dependencies—i.e., inheritance of centralized farming practices or late incorporation into EU technical assistance schemes—can link institutional adjustment to these, thus enhancing structural inefficiencies in food monitoring and hazard mitigation. On this score, enhancing food safety performance is more than technical adaptation; it entails long-term dedication to institution building, capacity building, and cultural adaptation to risk-based models of regulation.

## 6. Conclusions

The relative assessment demonstrates extensive deviation in the food safety record of EU member states across all three latest priority measures. Estonia, Lithuania, and Croatia topped the Fuzzy TOPSIS model with progressively few RASFF notifications, few pesticide maximum-residue-level (MRL) exceedances, and few per capita food losses. On the other hand, Bulgaria and Greece perennially suffered from high levels of MRL exceedance and RASFF alert levels and Greek food wastage rates. Notably, while some of the strict regulatory regime countries—like Finland and Luxembourg—have low MRL exceedances and moderate levels of RASFF, others, like Germany and Spain, rank mid-terrestrially based on average performance across indicators. Cluster analysis further classifies the countries into three typologies and identifies that, despite good monitoring, there are some countries that are plagued by issues related to sustainability-based indicators like food wastage. In totality, the findings utilize heterogeneity of national food safety profiles and performance complexity within EU countries.

These variations in food safety performance indicators are caused by an interplay of multifaceted institutional, economic, and cultural determinants. Northern and Western European countries such as Sweden, Finland, and the Netherlands possess superiorly equipped food safety systems, rigorous regulatory enforcement, and well-involved consumers to account for superior performance. These countries possess modern laboratory facilities, strict compliance with EU law, and openness of their population in the sense of lower MRL exceedances and good reporting under RASFF. Southern and Southeastern European countries, including Bulgaria, Greece, and Romania, on the contrary, tend to face structural barriers, i.e., weakly financed enforcement agencies, decentralized control of monitoring, and adherence to conventional farming methods. Cultural bent is also important; for example, where the consumer culture of food safety is deeply rooted and freely discussed in the public domain in a country, regulatory bodies are likely to be accountable and rightly focused on consumer interests. Second, food wastage that takes place to an undue degree in more advanced economies is more likely to be attributable to supply chain inefficiencies or over-consumption patterns rather than regulatory failure. All of these require food safety performance to be not just technically scoped, but institutional and cultural.

The overall findings of this study are of utility to inform food safety policy at both EU and national levels. Firstly, the direct connection between pesticide MRL exceedances and RASFF notifications reveals the immediate need for increased monitoring and strengthened regulation of pesticides, especially in lagging states. Second, because food waste was not perceived to contribute significantly to food safety notifications, food waste policy would be better included in targets for sustainability rather than for safety. At the EU level, differentiation policy clusters must be developed—i.e., high-risk countries must be supported with infrastructural support for laboratory upgrading, upgraded surveillance facilities, and the development of training capacity, and leading countries can serve as best-practice centers for diffusion. National action needs to combine regulation overhaul with national culture-sensitive public information campaigns in an attempt to influence consumer choice and build trust in food governance. Furthermore, ongoing investment in data integration and the provision of adaptive, multidimensional decision-support systems would facilitate more responsive and resilient food safety systems across the European Union. All in all, systemically based and context-aware coordination is the ticket to more effective and fairer food safety in the EU.

The key theoretical contribution of this paper lies in the fact that it constructs an integrative analytical framework to integrate food safety dimensions that were otherwise standalone—i.e., quantity of food safety notifications under RASFF, pesticide maximum-residue-level exceedance, and per capita wastage of food. Integrating these indicators into a composite comparative framework offers the research an alternative frame of reference that respects the interconnectedness of food safety monitoring regimes, agricultural practices, and consumers’ level behavior. This strategy transcends conventional, step-by-step assessment and underscores the need to embrace a systemic perspective—the multilateral and dynamic character of food safety in the EU’s member states.

Through the use of decision-making methods grounded in fuzzy logic (Fuzzy TOPSIS) combined with traditional statistical methods, this study presents a novel approach that is responsive to food safety data heterogeneity and uncertainty. This nuanced use of fuzzy clustering and scenario-based sensitivity analysis enhances current theoretical models through the recognition of partial and sometimes fuzzy membership of countries to several performance profiles. Thus, this study not only plugs one of the most significant gaps in the literature—few have synthesized these disparate strands of food safety governance—but it lays a foundation for subsequent research that can develop this through a learning, evidence-based framework.

The policy conclusions of this paper can be instantiated directly toward European Union policy makers, regulatory agencies, and agri-food industry stakeholders. Exceedances of pesticide MRL are identified as the most significant driver to RASFF notifications by this study, providing direct, evidence-based justification for increasing phytosanitary controls and investment in State-of-the-Art laboratory facilities in risk countries. The clustering analysis also allows differentiated policy targeting, where interventions and resources can be aligned with the individual risk profile of each country instead of blanket regulation. In addition, the incorporation of scenario-based sensitivity analysis enables decision-makers to possess a forecasting tool for assessing the potential impacts of policy measures, for example, lowering pesticide exceedance levels or removing food waste inefficiencies. Collectively these results present an integrated working template for raising the target of food safety governance while sustaining a wider commitment to consumer protection and sustainability within the EU food system.

This study is limited by the cross-sectional design, relying on data only, and these data may not fully capture long-term trends or cyclical fluctuations in food safety measures. Moreover, the analysis is also limited by the quality and availability of data among the EU member states, differing with respect to national reporting systems and administrative capacity.

Follow-up studies could then capitalize on these results by using longitudinal data to estimate long-term change in food safety performance and identify emerging trends. It would be helpful, as well, to incorporate qualitative methods—e.g., expert interviews or surveys of stakeholders—to add richness to the analysis of issues with policy uptake and local contexts.

## Figures and Tables

**Table 1:** Food safety indicators in EU countries for 2022.

Country	RASFF Notifications (2022)	Pesticide MRL Exceedances (%)	Food Waste (kg/Person/Year)
Austria	45	0.80%	133
Belgium	60	1.20%	129
Bulgaria	212	1.50%	94
Croatia	30	0.70%	76
Cyprus	25	0.90%	96
Czech Republic	40	1.00%	91
Denmark	50	0.60%	135
Estonia	20	0.50%	86
Finland	35	0.40%	137
France	100	1.10%	132
Germany	100	1.00%	130
Greece	80	1.30%	142
Hungary	45	1.00%	90
Ireland	25	0.50%	135
Italy	90	1.20%	129
Latvia	18	0.70%	88
Lithuania	15	0.60%	85
Luxembourg	10	0.40%	139
Malta	12	0.80%	97
Netherlands	70	0.90%	134
Poland	60	1.20%	106
Portugal	55	1.10%	131
Romania	50	1.30%	95
Slovakia	35	0.90%	89
Slovenia	20	0.60%	92
Spain	90	1.40%	128
Sweden	40	0.50%	136

Source: On basis of data from [99,100,101].

**Table 2:** Food safety indicators in EU countries for 2020.

Country	RASFF Notifications (2020)	Pesticide MRL Exceedances (%)	Food Waste (kg/Person/Year)
Austria	267	0.80%	133
Belgium	215	1.20%	129
Bulgaria	21	1.50%	94
Croatia	16	0.70%	76
Cyprus	70	0.90%	96
Czech Republic	105	1.00%	91
Denmark	27	0.60%	135
Estonia	81	0.50%	86
Finland	220	0.40%	137
France	531	1.10%	132
Germany	51	1.00%	130
Greece	28	1.30%	142
Hungary	58	1.00%	90
Ireland	297	0.50%	135
Italy	32	1.20%	129
Latvia	103	0.70%	88
Lithuania	31	0.60%	85
Luxembourg	4	0.40%	139
Malta	498	0.80%	96
Netherlands	185	0.90%	134
Poland	30	1.20%	106
Portugal	33	1.10%	131
Romania	35	1.30%	93
Slovakia	40	0.90%	89
Slovenia	193	0.60%	92
Spain	115	1.40%	128
Sweden	267	0.50%	136

Source: On basis of data from [99,100,101].

**Table 3:** Ranking of EU countries according to the Fuzzy TOPSIS method.

Country	Closeness	Rank
Estonia	0.8951	1
Lithuania	0.8706	2
Croatia	0.8406	3
Slovenia	0.8267	4
Latvia	0.8152	5
Malta	0.7378	6
Slovakia	0.7192	7
Cyprus	0.6991	8
Czech Republic	0.6699	9
Hungary	0.6682	10
Luxembourg	0.5972	11
Ireland	0.5908	12
Finland	0.5882	13
Sweden	0.5743	14
Denmark	0.5521	15
Romania	0.5501	16
Austria	0.5247	17
Poland	0.5204	18
Netherlands	0.4650	19
Portugal	0.4492	20
Belgium	0.4258	21
Germany	0.4066	22
Italy	0.3747	23
France	0.3716	24
Spain	0.3380	25
Greece	0.3365	26
Bulgaria	0.3355	27

**Table 4:** Detailed clustering results of EU countries.

Country	Cluster	RASFF_Norm	MRL_Norm	Food-Waste_Norm
Austria	2	0.1733	0.3636	0.8636
Belgium	0	0.2475	0.7273	0.8030
Bulgaria	0	1.0000	1.0000	0.2727
Croatia	1	0.0990	0.2727	0.0000
Cyprus	1	0.0743	0.4545	0.3030
Czech Republic	1	0.1485	0.5455	0.2273
Denmark	2	0.1980	0.1818	0.8939
Estonia	1	0.0495	0.0909	0.1515
Finland	2	0.1238	0.0000	0.9242
France	0	0.4455	0.6364	0.8485
Greece	0	0.3465	0.8182	1.0000
Spain	0	0.3960	0.9091	0.7879
Netherlands	0	0.2970	0.4545	0.8788
Ireland	2	0.0743	0.0909	0.8939
Lithuania	1	0.0248	0.1818	0.1364
Luxembourg	2	0.0000	0.0000	0.9545
Latvia	1	0.0396	0.2727	0.1818
Malta	1	0.0099	0.3636	0.3182
Germany	0	0.4455	0.5455	0.8182
Poland	0	0.2475	0.7273	0.4545
Portugal	0	0.2228	0.6364	0.8333
Romania	1	0.1980	0.8182	0.2879
Slovakia	1	0.1238	0.4545	0.1970
Slovenia	1	0.0495	0.1818	0.2424
Sweden	2	0.1485	0.0909	0.9091
Hungary	1	0.1733	0.5455	0.2121
Italy	0	0.3960	0.7273	0.8030

**Table 5:** Correlation matrix for 2022.

	RASFF Notifications	Pesticide MRL Exceedances (%)	Food Waste (kg/Person/Year)
RASFF notifications	1.000 (*p* = 0.0000)	0.722 (*p* = 0.0000)	0.200 (*p* = 0.3183)
Pesticide MRL (%)	0.722 (*p* = 0.0000)	1.000 (*p* = 0.0000)	0.003 (*p* = 0.9887)
Food waste (kg/year)	0.200 (*p* = 0.3183)	0.003 (*p* = 0.9887)	1.000 (*p* = 0.0000)

**Table 6:** Correlation matrix 2020.

	RASFF Notifications	Pesticide MRL Exceedance (%)	Food Waste (kg/Person/Year)
RASFF notifications	1.000 (*p* = 0.0000)	−0.962 (*p* = 0.0000)	−0.524 (*p* = 0.1202)
Pesticide MRL exceedance (%)	−0.962 (*p* = 0.0000)	1.000 (*p* = 0.0000)	0.703 (*p* = 0.0233)
Food waste (kg/person/year)	−0.524 (*p* = 0.1202)	0.703 (*p* = 0.0233)	1.000 (*p* = 0.0000)

**Table 7:** Multiple linear regression results for 2022.

Parameter	Coefficient	Std. Error	t-Statistic	*p*-Value	95% CI Lower	95% CI Upper
Intercept	−72.561	33.096	−2.192	0.038	−140.868	−4.254
Pesticide MRL exceedances (%)	93.967	17.656	5.322	0.000	57.526	130.407
Food waste (kg/person/year)	0.369	0.253	1.458	0.158	−0.153	0.891

**Table 8:** Multiple linear regression results for 2020.

Parameter	Regression Coefficient	Standard Error	t-Statistic	*p*-Value	95% Confidence Interval (Lower)	95% Confidence Interval (Upper)
Intercept	135.429	45.762	2.958	0.008	40.868	229.991
Pesticide MRL exceedances (%)	−112.508	23.443	−4.798	0.000	−160.857	−64.158
Food waste (kg/person/year)	−0.773	0.312	−2.479	0.021	−1.423	−0.122

**Table 9:** Linear regression results for 2020 (dependent variable: number of RASFF notifications).

Parameter	Coefficient	Standard Error	t-Statistic	*p*-Value	95% Confidence Interval (Lower)	95% Confidence Interval (Upper)
Intercept	135.429	45.762	2.958	0.008	40.868	229.991
Pesticide MRL exceedances (%)	−112.508	23.443	−4.798	0.000	−160.857	−64.158

**Table 10:** Linear regression results for 2022 (dependent variable: number of RASFF notifications).

Parameter	Coefficient	Standard Error	t-Statistic	*p*-Value	95% Confidence Interval (Lower)	95% Confidence Interval (Upper)
Intercept	−30.902	17.077	−1.810	0.082	−66.074	4.269
Pesticide MRL exceedances (%)	94.040	18.049	5.210	0.000	56.867	131.213

**Table 11:** Sensitivity analysis results of 2022.

Variable	Coefficient	Mean of Variable	Semi-Elasticity
Pesticide MRL exceedances (%)	93.967	0.893	105.274
Food waste (kg/person/year)	0.369	113.148	0.003

**Table 12:** Sensitivity analysis results for 2020.

Variable	Coefficient	Mean of Variable	Semi-Elasticity
Pesticide MRL exceedances (%)	−112.508	0.893	−100.423
Food waste (kg/person/year)	−0.773	113.148	−0.0068

**Table 13:** Sensitivity analysis of reduced models.

Year	MRL Coefficient	Mean MRL (%)	Semi-Elasticity
2020	−77.74	0.893	−87.10
2022	94.04	0.893	105.36

**Table 14:** Scenario analysis results.

Scenario	Predicted RASFF Notifications
Baseline	53.037
+10% MRL	61.424
−10% MRL	44.650
+10% Food waste	57.209
−10% Food waste	48.865

## Data Availability

The original contributions presented in this study are included in the article. Further inquiries can be directed to the corresponding author.
